# Increased biventricular hemodynamic forces in precapillary pulmonary hypertension

**DOI:** 10.1038/s41598-022-24267-6

**Published:** 2022-11-19

**Authors:** Karin Pola, Elsa Bergström, Johannes Töger, Göran Rådegran, Per M. Arvidsson, Marcus Carlsson, Håkan Arheden, Ellen Ostenfeld

**Affiliations:** 1 Clinical Physiology, Department of Clinical Sciences Lund, Lund University, Skåne University Hospital, Lund, Sweden; 2 Department of Clinical Sciences Lund, Cardiology, and Skåne University Hospital, Section of Heart Failure and Valvular Disease, Lund University, Lund, Sweden

**Keywords:** Physiology, Cardiovascular biology, Circulation, Cardiology, Cardiovascular biology, Medical research

## Abstract

Precapillary pulmonary hypertension (PH_precap_) is a condition with elevated pulmonary vascular pressure and resistance. Patients have a poor prognosis and understanding the underlying pathophysiological mechanisms is crucial to guide and improve treatment. Ventricular hemodynamic forces (HDF) are a potential early marker of cardiac dysfunction, which may improve evaluation of treatment effect. Therefore, we aimed to investigate if HDF differ in patients with PH_precap_ compared to healthy controls. Patients with PH_precap_ (n = 20) and age- and sex-matched healthy controls (n = 12) underwent cardiac magnetic resonance imaging including 4D flow. Biventricular HDF were computed in three spatial directions throughout the cardiac cycle using the Navier–Stokes equations. Biventricular HDF (N) indexed to stroke volume (l) were larger in patients than controls in all three directions. Data is presented as median N/l for patients vs controls. In the RV, systolic HDF diaphragm-outflow tract were 2.1 vs 1.4 (p = 0.003), and septum-free wall 0.64 vs 0.42 (p = 0.007). Diastolic RV HDF apex-base were 1.4 vs 0.87 (p < 0.0001), diaphragm-outflow tract 0.80 vs 0.47 (p = 0.005), and septum-free wall 0.60 vs 0.38 (p = 0.003). In the LV, systolic HDF apex-base were 2.1 vs 1.5 (p = 0.005), and lateral wall-septum 1.5 vs 1.2 (p = 0.02). Diastolic LV HDF apex-base were 1.6 vs 1.2 (p = 0.008), and inferior-anterior 0.46 vs 0.24 (p = 0.02). Hemodynamic force analysis conveys information of pathological cardiac pumping mechanisms complementary to more established volumetric and functional parameters in precapillary pulmonary hypertension. The right ventricle compensates for the increased afterload in part by augmenting transverse forces, and left ventricular hemodynamic abnormalities are mainly a result of underfilling rather than intrinsic ventricular dysfunction.

## Introduction

Precapillary pulmonary hypertension (PH_precap_) is a vascular condition encompassing several diseases with adverse outcome^1^. Pulmonary arterial hypertension (PAH) and chronic thromboembolic pulmonary hypertension (CTEPH) are subgroups of PH_precap_ that exhibit common features of elevated pulmonary arterial pressure and vascular resistance, without an underlying pulmonary or left ventricular heart disease^1^. The increased pulmonary vascular resistance leads to increased pressure and influences cardiac morphology and function, leading to right heart failure and impaired filling of the left side of the heart and to premature mortality^1–4^. Alterations in pumping mechanisms may occur with preserved ventricular volumes, and current methods for monitoring treatment effect and deterioration are imprecise^2,5,6^.

Hemodynamic forces (HDF) computed from cardiac magnetic resonance imaging (CMR) have the potential to be used as a marker of cardiac dysfunction^7–9^, which may improve evaluation of treatment effect in patients with PH_precap_. Pressure gradients are created within the ventricle by ventricular contraction and relaxation, which result in hemodynamic forces causing the blood to accelerate^7,10^. Intracardiac HDF can be computed using a reference standard CMR method with 4D flow data, where blood flow is measured in all three spatial directions and over time^11,12^.

Changes in right (RV) and left (LV) ventricular volumes in patients may impact HDF, as force equals mass multiplied by acceleration. However, ventricular pumping and filling mechanisms could alter HDF even with preserved end-diastolic volume and ejection fraction, as HDF are a measure of the three-dimensional acceleration of blood. Measuring HDF over time can thus quantify the impulses causing blood to flow, which may differ between ventricles with the same volumes. Patients with PH_precap_ typically have a remodeled right ventricular anatomical shape and a pump physiology with lower LV volumes and altered LV regional contribution to stroke volume (SV), but preserved LV ejection fraction^3,5,13,14^. Previous studies have shown altered HDF in patients with volume-overloaded ventricles^15,16^. Ventricular HDF may therefore have the potential to be a more sensitive marker of pathological cardiac pumping mechanisms than typical volumetric and functional parameters in this patient cohort^7,8,17,18^, which may improve evaluation of treatment effect. The aim of this study was therefore to investigate if hemodynamic force patterns in patients with precapillary pulmonary hypertension differ compared to healthy controls.

## Methods

This observational study included patients being investigated for suspected precapillary pulmonary hypertension (PH_precap_) at our tertiary PH center where CMR was performed as part of the routine evaluation, and healthy controls as a reference. Recruitment and data acquisition took place between 2016 and 2021. The patient inclusion criteria were confirmed diagnosis of PH_precap_ owing to either pulmonary arterial hypertension (PAH) or chronic thromboembolic pulmonary hypertension (CTEPH).

Ten healthy participants from the ongoing population-based study SCAPIS (Swedish CArdioPulmonary bioImage Study)^19^ were included as a control group after previous participation in a baseline exam, and underwent an additional examination with CMR and 4D flow. Two healthy controls were included from previous studies in our group. The controls were matched to the patient cohort with a group-level comparison for age and sex. The SCAPIS baseline exam included coronary computed tomography, ultrasound of the carotid arteries, and spirometry as well as clinical labs and subject history. Exclusion criteria for the healthy subjects were any previously known systemic or cardiovascular disease or any pathology discovered at the imaging examinations, use of cardiovascular medications, smoking, systemic blood pressure > 140/90 mmHg, or any pathology at electrocardiography or CMR.

Ethical approval was obtained from the Ethical Review Board, Lund, Sweden (application numbers 2010/114, 2010/248, 2011/777), and all participants gave written informed consent to participate prior to examination. The study was conducted in accordance with the Helsinki Declaration and reported in accordance with the STROBE recommendations^20^.

### Right heart catheterization

Patients underwent clinically indicated right heart catheterization to confirm the diagnosis of PH_precap_. A triple-lumen Swan-Ganz catheter was used to measure mean pulmonary arterial pressure (mPAP), pulmonary artery wedge pressure (PAWP), right atrial pressure, cardiac output (CO) and pulmonary vascular resistance (PVR = (mPAP-PAWP)/CO). Measurements were obtained during free breathing in supine position with local anesthesia and were acquired as a mean value over several heart beats. PH_precap_ was defined by mPAP ≥ 25 mmHg at rest with PAWP ≤ 15 mmHg and PVR > 3 Wood Units according to the prevailing European Society of Cardiology/European Respiratory Society guidelines at the time of the study^1^. Systemic vascular resistance was calculated in Wood Units using the following formula^21^:

$$Systemic\, vascular\, resistance={\frac{mean\, arterial\, pressure- mean\,right\, \,atrial\, pressure}{cardiac\, output}}$$

Mean arterial pressure was calculated non-invasively as follows: Diastolic pressure + 1/3(systolic−diastolic pressure). Mean right atrial pressure was measured invasively in patients and estimated to 3 mmHg in controls. Cardiac output was measured from CMR.

### CMR image acquisition

Cardiovascular magnetic resonance images were acquired at 1.5 T (MAGNETOM Aera, Siemens Healthcare, Erlangen, Germany). The protocol consisted of standard short- and long-axis (2-, 3- and 4-chamber view) cine balanced steady-state free precession images, as well as three-dimensional, time-resolved phase contrast flow (4D flow) images sampled from a box covering the heart and proximal great vessels using a prototype 4D flow sequence^22–26^. Typical 4D flow sequence parameters were as follows: acquired temporal resolution 46 ms, acquired spatial resolution 3 mm isotropic, acceleration methods GRAPPA factor 3–4 (3 × 1 or 2 × 2, phase × slice encoding direction) and partial Fourier 6/8 in both phase encoding and slice encoding directions. Detailed sequence parameters are shown in Supplementary Table 1. The 4D flow data sets were corrected for phase background errors using fitting to stationary tissue, and based on a visual determination either first- or second order correction for each subject^27,28^. Aliasing errors were corrected by automatic phase unwrapping with manual adjustments^29^.

Stroke volume measured with 4D flow has previously been validated against 2D flow in healthy subjects and laser-based reference methods in phantoms^11,24,30^. However, 4D flow measurements could have lower accuracy and precision in patients with right heart failure, as patients often have larger gross body motion and respiratory motion during the scan, which may result in motion artifacts. To ensure adequate data quality, we therefore performed a validation of flow volumes in 4D flow with 2D flow as the reference standard (Appendix). Flow was analyzed in patients and controls in the ascending and descending aorta, main pulmonary artery, superior vena cava and right inferior pulmonary vein (Appendix Fig. 1). Net flow during one cardiac cycle was computed from both 4D and 2D measurements in all delineated vessels (Appendix Table 1 and Appendix Figs. 1 and 2).

### Data analysis

Image analysis was performed using the software Segment v2.2 R7052 (http://segment.heiberg.se)^31^.

Biventricular volumes and mass were defined by semi-automatic delineation of the RV and LV endo- and epicardium in short-axis cine CMR images for the entire cardiac cycle, including papillary muscles and trabeculations to the ventricular volumes according to recommendations^32^. Stroke volume (SV) was calculated for each ventricle as the difference between end-diastolic and end-systolic volumes, and ejection fraction (EF) as SV divided by end-diastolic volume. Myocardial mass was computed as the difference between epi- and endocardial volumes multiplied by an assumed myocardial density of 1.05 g/ml. Atrioventricular plane displacement (AVPD) was measured in mm as the difference of AV-plane position from end diastole to end systole, as previously described^33,34^. Peak longitudinal strain was measured in the RV and LV using manual delineations of the myocardium in end diastole in the 4-chamber long-axis view, using feature tracking in Segment v2.2 R7052.

### Computation of hemodynamic forces

**Figure 1:**
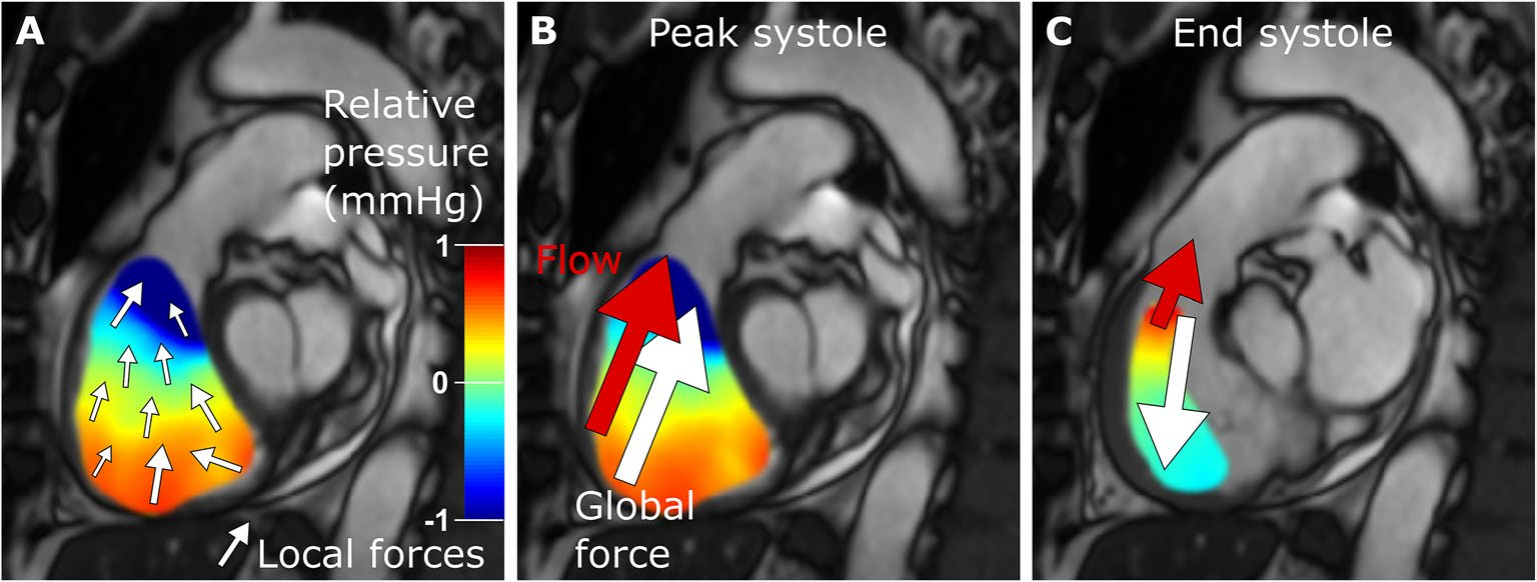
Right ventricular outflow tract in one patient with precapillary pulmonary hypertension: relative pressure gradients, hemodynamic forces and flow. (**A**) The colored field illustrates the relative pressure gradients. Local hemodynamic forces are illustrated with white arrows, with direction and magnitude indicated for each point. (**B**) The global force (white arrow) is a sum of all local forces and drives the blood flow (red arrow) towards the pulmonary artery during systole. (**C**) By the end of systole, the global force is directed in the opposite direction of the flow, thereby decelerating the blood. The global force is analyzed in three directions for each ventricle (Fig. 2).

**Figure 2:**
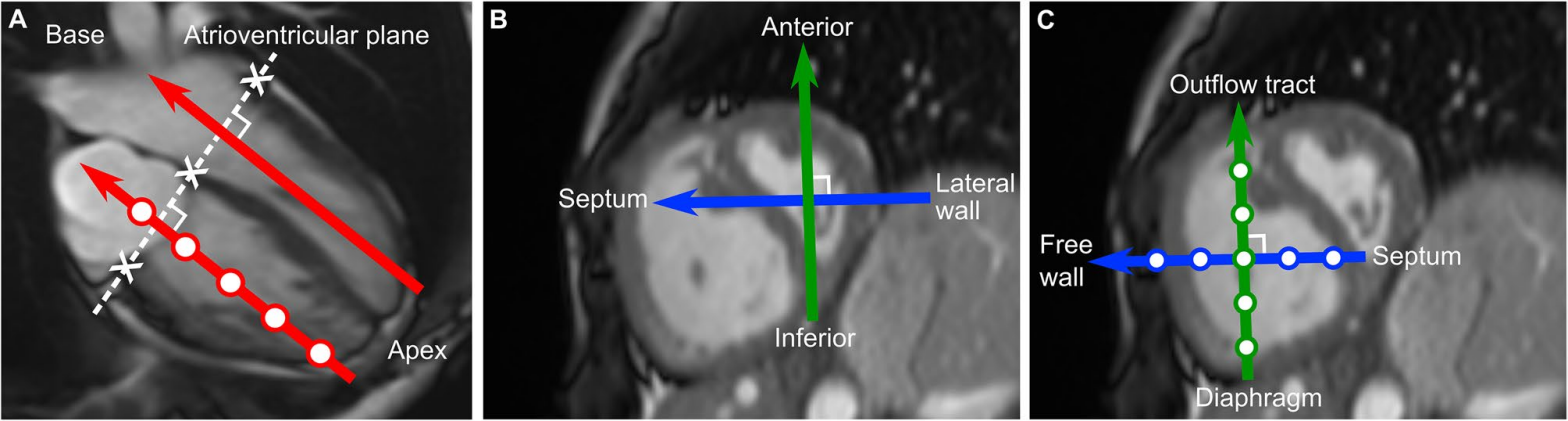
Definition of the intraventricular three-dimensional directions, using an orientation system previously described^7^. (**A**) Semi-automatic definition of the atrioventricular (AV) plane in end diastole by manually marking the AV junction points (white crosses) in the long-axis views, and thereafter an automatic construction of a two-dimensional plane fitted to the points. The apex-base direction is defined as perpendicular to the AV-plane. (**B**) and (**C**) The lateral wall-septum direction is perpendicular to the apex-base axis, and aligned with the left ventricular outflow tract in the 3-chamber long-axis image. The inferior-anterior direction is perpendicular to both the apex-base and lateral wall-septum directions. The transverse directions were defined from the left ventricle (**B**) and translated to the right ventricle (**C**), resulting in parallel interventricular directions.

Intraventricular hemodynamic forces were quantified using a validated method previously described in detail^7^. In summary, pressure gradients from 4D-flow data were computed using the Navier–Stokes equations and integrated over the RV and LV cavities defined by delineations in the short-axis cine images. Hemodynamic forces were calculated from the field of pressure gradients (Fig. 1), and the intraventricular directions of the force vectors were defined using a spatial reference system originating from the position of the AV plane (Fig. 2). The AV plane was defined by eight points manually placed in the three long-axis views, and the apex-base direction was set as perpendicular to the AV plane. The LV lateral wall-septum direction was set as perpendicular to the apex-base direction and parallel to the 3-chamber view. The LV inferior-anterior direction was set as perpendicular to both the apex-base and the lateral wall-septum direction. The intraventricular directions of the RV are identical to the LV reference system, but axes were renamed to reflect the RV anatomical landmarks.

To enable comparison of hemodynamic forces between hearts with varying duration of the cardiac cycle, a common time axis was created by linear resampling of the force curves, using end diastole and end systole as temporal landmarks. End diastole was defined as the largest ventricular volume in the short-axis images. End systole was defined from the flow curves derived from 4D data of the blood flow in the aorta for the LV and the pulmonary artery for the RV. By linear extrapolation of the systolic downslope between 75% and 25% of maximum amplitude, end systole was determined as the time point on the extrapolated line where the amplitude reached zero (Supplementary Fig. 1)^7,11^.

Root mean square (RMS) analysis was used to facilitate comparison of force magnitudes in the three directions regardless of vector direction, and as a method to compare cardiac phases with large standard deviations or no clear peaks within the groups, as in previous studies^7,9^. Peak forces were analyzed for systole and diastole in all three directions as a quantitative measurement of the HDF patterns during the cardiac cycle. Hemodynamic forces were indexed to SV and associations between HDF and SV were analyzed to investigate whether differences in volumes could explain differences in HDF between patients and controls. Force ratio was calculated in the RV and LV for systole and diastole as the proportion of transversal to longitudinal forces, as a quantitative measurement of the principal direction of HDF in patients compared to controls. Hemodynamic force ratio was calculated as a vector sum of the two transversal components divided by the longitudinal component, using the following formula:

$$\text{RV force ratio} = {\frac{\sqrt{{\text{Septum-Free wall}}^{2}+{\text{Diaphragm-Outflow}\, \text{tract}}^{2}}}{\text{Apex-Base}}},$$

$$\text{LV force ratio} = {\frac{\sqrt{{\text{Lateral wall-Septum}}^{2}{+\text{Inferior-Anterior}}^{2}}}{\text{Apex-Base}}}.$$

### Statistical analysis

Statistical analyses were performed using GraphPad Prism v9.3.1 (GraphPad Software, La Jolla, California, USA). Continuous data is presented as median and interquartile range [IQR] and categorical data as absolute numbers and proportion (%). The Mann–Whitney U test was used to compare continuous data between groups, and Fisher’s exact test was used to compare binary categorical data. Associations between HDF and SV were analyzed using linear regression analysis and coefficient of determination (R^2^). A two-tailed p-value < 0.05 was considered significant.

## Results

### Patient characteristics

Out of 44 patients investigated for PH_precap_ and examined with CMR 4D flow, 24 patients were excluded, due to inadequate 4D-data quality (n = 7), missing cine slices (n = 1), congenital heart disease (n = 5), PH due to lung disease (group 3, n = 3), PH due to LV disease (group 2, n = 1), or no PH diagnosis (n = 7). Twenty patients (16 women, 69 [18] years) and 12 controls (9 women, 62 [6] years) were included in the final analyses.

All patients fulfilled the criteria of precapillary pulmonary hypertension, having mPAP 41 [18] mmHg, PAWP 8 [3] mmHg and PVR 7 [5] Wood units (Table 1). Patients had comorbidities typical for a PH_precap_ cohort^35^, which were clinically assessed as not causative to PH. Thirty percent of patients were on PH-specific medical treatment at the time of CMR. There were no statistically significant differences between patients and controls in sex, body mass index, body surface area, cardiac output, cardiac index, RV SV, LV EF, or LV mass (Table 2). Patients had larger RV mass and end-systolic volume, lower RV EF and RV AVPD, smaller LV end-diastolic volumes and LV SV, and lower LV AVPD compared to controls. Patients with PH_precap_ had decreased RV longitudinal strain compared to controls (20% [4.3] vs 24% [4.5], p = 0.006), but there was no difference between the groups in LV longitudinal strain (18% [4.9] vs 19% [2.9], p = 0.6).

**Table 1:** Subject characteristics of patients with precapillary pulmonary hypertension.

PH_precap_ (n = 20)	
**PH**_**precap**_** subgroups**	
Pulmonary arterial hypertension	13 (65%)
Chronic thromboembolic pulmonary hypertension	7 (35%)
Incidental cases at CMR	14 (70%)
NT-proBNP	475 [1332]
**Right heart catheterization**	
Systolic pulmonary arterial pressure (mmHg)	70 [31]
Mean pulmonary arterial pressure (mmHg)	41 [18]
Right ventricular pressure (mmHg)	
- Systole	68 [31]
- Diastole	0.5 [5]
- End diastole	9 [4]
Mean right atrial pressure (mmHg)	5 [5]
Pulmonary artery wedge pressure (mmHg)	8 [3]
Pulmonary vascular resistance (Wood units)	7 [5]
**Comorbidities**	
Atrial fibrillation	0 (0%)
Chronic Obstructive Pulmonary Disease/Emphysema	6 (30%)
Diabetes	5 (25%)
Ischemic heart disease	2 (10%)
Systemic hypertension	9 (45%)
Right bundle branch block, complete or incomplete	2 (10%)
Left bundle branch block	1 (5%)
**Medication at CMR**	
PH-specific medication	
- Single therapy	2 (10%)
- Dual therapy	2 (10%)
- Triple therapy	2 (10%)
Other medications	
- Calcium channel blocker	6 (30%)
- ACEi/ARB/ARNI	8 (40%)
- Betablocker	5 (25%)
- Diuretics	8 (40%)

Data is expressed as median and interquartile range [IQR] or absolute numbers and proportion (%).

ACEi, angiotensin-converting enzyme inhibitor; ARB, angiotensin receptor blocker; ARNI, angiotensin receptor-neprilysin inhibitor; CMR, cardiovascular magnetic resonance imaging; PH_precap_, precapillary pulmonary hypertension. PH-specific medication includes endothelin receptor antagonist, phosphodiesterase 5 inhibitor, prostaglandin analogue, selective prostacyclin receptor agonist, soluble guanylate cyclase stimulator.

**Table 2:** Cardiac volumes and functional measures from CMR*.*

	PH_precap_ (n = 20)	Controls (n = 12)	p-value
**Subject characteristics**			
Age (years)	69 [18]	62 [6]	0.09
Women, n (%)	16 (80)	9 (75)	1
Body mass index (kg/m^2^)	26 [6.7]	25 [3.1]	0.09
Body surface area (m^2^)	1.8 [0.22]	1.8 [0.26]	1
Non-invasive systemic blood pressure (mmHg)			
- Systole	138 [29]	122 [17]	*0.048*
- Diastole	83 [21]	77 [14]	0.1
Systemic vascular resistance (Wood Units)	21 [12]	18 [5.1]	0.2
**CMR measurements**			
Heart rate (bpm)	76 [15]	69 [10]	*0.03*
RV EDV (ml)	184 [92]	148 [41]	0.08
RV ESV (ml)	116 [74]	71 [16]	*0.009*
RV SV (ml)	72 [32]	77 [25]	0.4
RV EF (%)	40 [21]	53 [4.3]	*0.002*
RV AVPD (mm)	14 [3.4]	18 [4.2]	*0.0003*
RV mass (g)	32 [17]	17 [6.3]	*0.0002*
LV EDV (ml)	125 [52]	151 [33]	*0.03*
LV ESV (ml)	61 [18]	70 [19]	0.07
LV SV (ml)	61 [24]	84 [21]	*0.02*
LV EF (%)	54 [11]	54 [4.3]	0.7
LV AVPD (mm)	11 [3.5]	14 [1.9]	*0.01*
LV mass (g)	71 [13]	62 [27]	0.9
Cardiac output (l/min)	4.3 [2.4]	5.4 [1.5]	0.2
Cardiac index (l/min/m^2^)	2.5 [1.1]	3.0 [0.5]	0.3

Significant values are in [italics].

Data is expressed as median and interquartile range [IQR].

PH_precap_, precapillary pulmonary hypertension; CMR, cardiac magnetic resonance; bpm, beats per minute; RV EDV, right ventricular end-diastolic volume; ESV, end-systolic volume; SV, stroke volume; EF, ejection fraction; AVPD, atrioventricular plane displacement; LV, left ventricular.

### Hemodynamic forces, right ventricle

**Figure 3:**
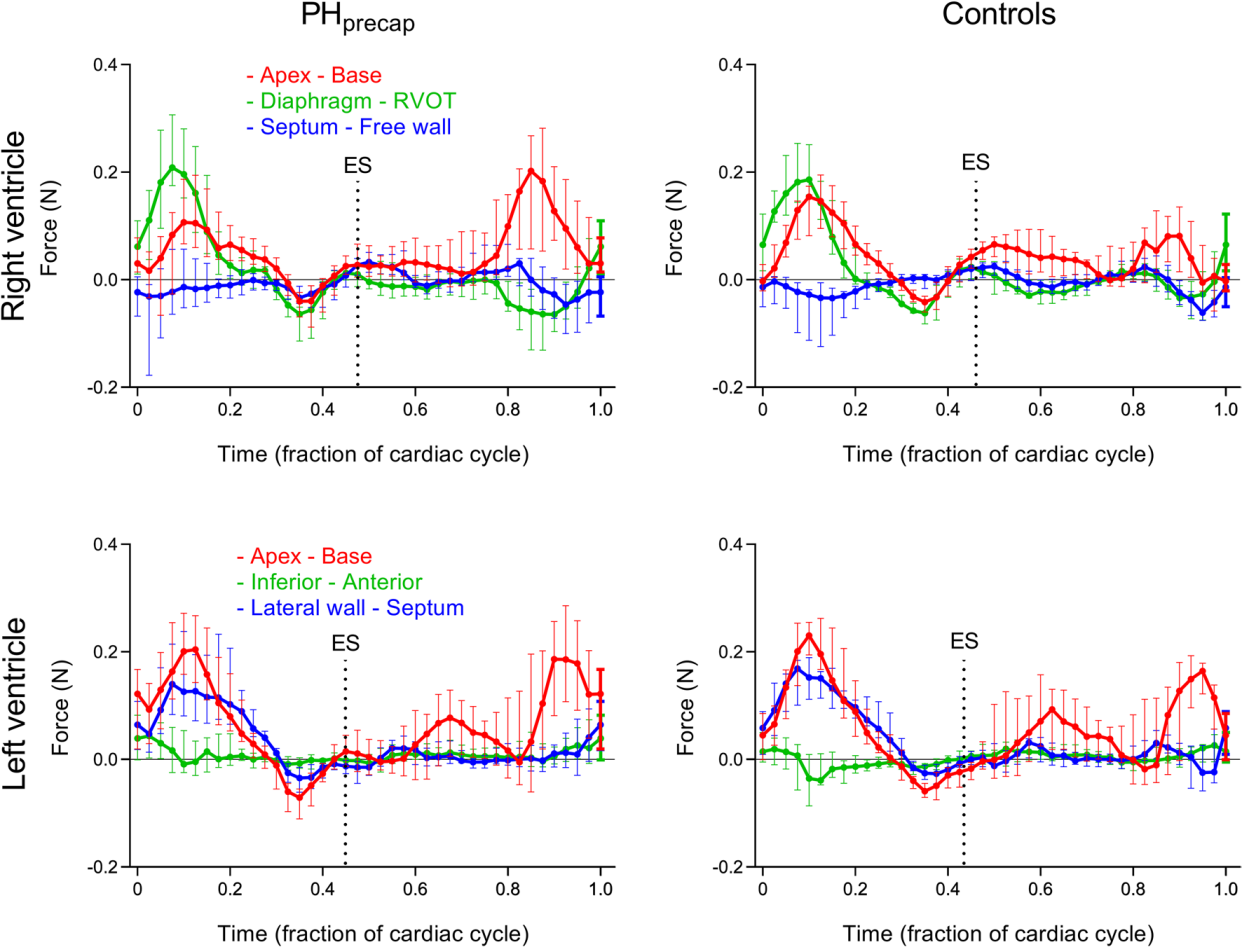
Right (top row) and left (bottom row) ventricular hemodynamic forces over one cardiac cycle in patients with precapillary pulmonary hypertension (PH_precap_, left column) compared to healthy controls (right column). Force patterns are presented as median and interquartile range for each time point in all three directions respectively. Individual force curves for patients and controls are shown in Supplementary Figs. 2 (RV) and 6 (LV). ES, end systole; RVOT, right ventricular outflow tract.

Right ventricular force patterns over one cardiac cycle are presented as median and interquartile range for each time point in the three orthogonal directions in Fig. 3 for patients and controls. Individual force curves are presented in Supplementary Fig. 2.

#### RV hemodynamic forces indexed to stroke volume

**Figure 4:**
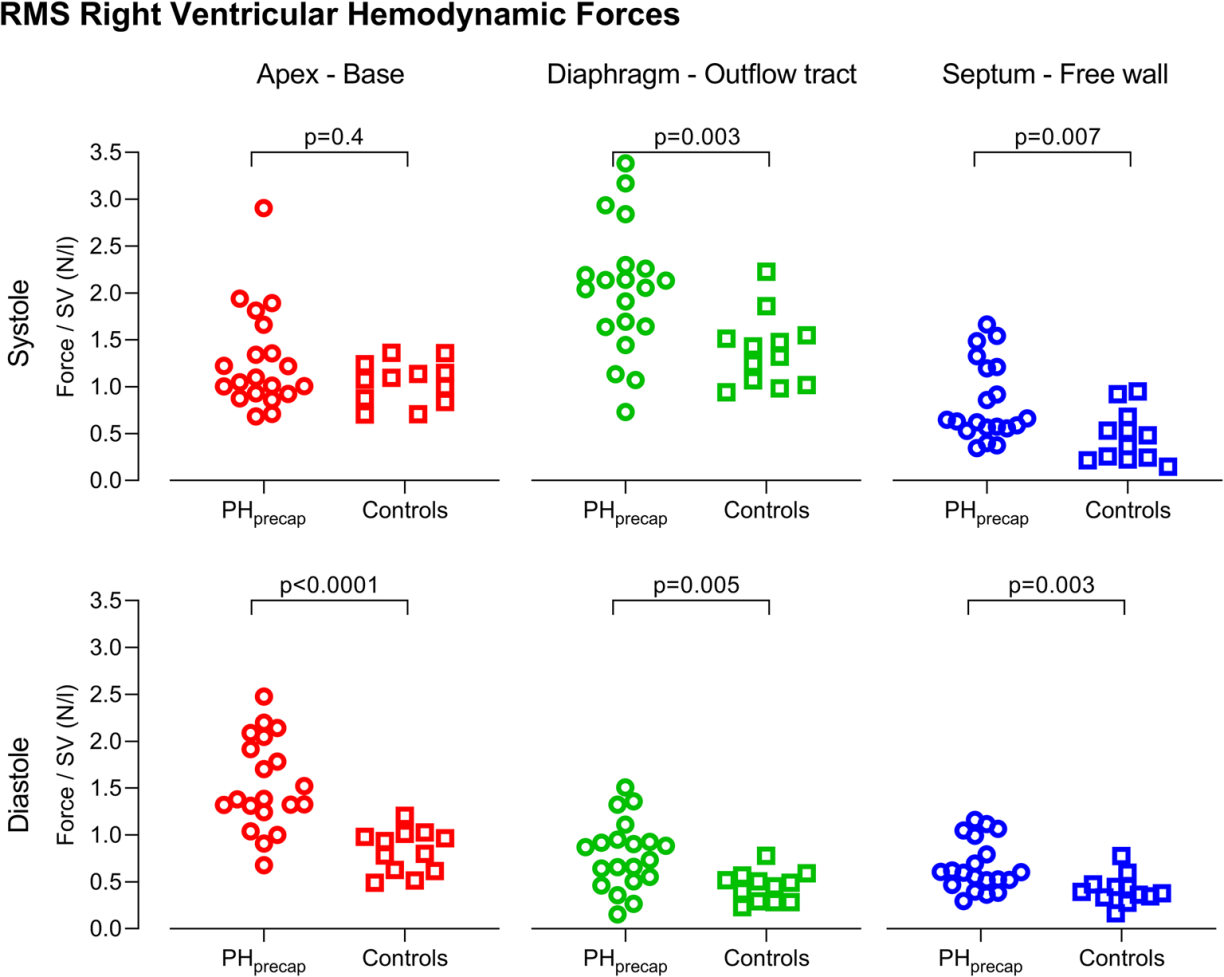
Root mean square (RMS) right ventricular hemodynamic forces in patients with precapillary pulmonary hypertension (PH_precap_, circles) compared to healthy controls (squares). RMS hemodynamic forces indexed to right ventricular stroke volume (SV) during systole (top row) and diastole (bottom row), in the apex-base direction (left column, red), diaphragm-outflow tract direction (middle column, green), and septum-free wall direction (right column, blue).

Right ventricular HDF indexed to SV is presented in Fig. 4 and Supplementary Table 2 for RMS forces and in Supplementary Fig. 3 and Supplementary Table 3 for peak forces. Patients had larger HDF (both RMS and peak) than controls in systole in the diaphragm-outflow tract and in the septum-free wall directions and in diastole in all three directions.

#### RV hemodynamic forces in absolute values

RV HDF in absolute values is presented in Supplementary Fig. 4 and Supplementary Table 4 for RMS forces and Supplementary Fig. 5 and Supplementary Table 5 for peak forces. In systole, peak HDF was larger in patients than controls in the septum-free wall direction. In diastole, patients had larger forces (both RMS and peak) than controls in the apex-base direction, but no difference was found between the groups in any other direction.

#### RV force ratio

The ratio between RV transverse and longitudinal hemodynamic forces did not differ between the groups in systole or diastole (p = 0.07 for systole and p = 0.3 for diastole).

### Hemodynamic forces, left ventricle

Left ventricular force patterns over one cardiac cycle are presented as median and interquartile range for each time point in the three orthogonal directions in Fig. 3 for patients and controls. Individual force curves are presented in Supplementary Fig. 6.

#### LV hemodynamic forces indexed to stroke volume

**Figure 5:**
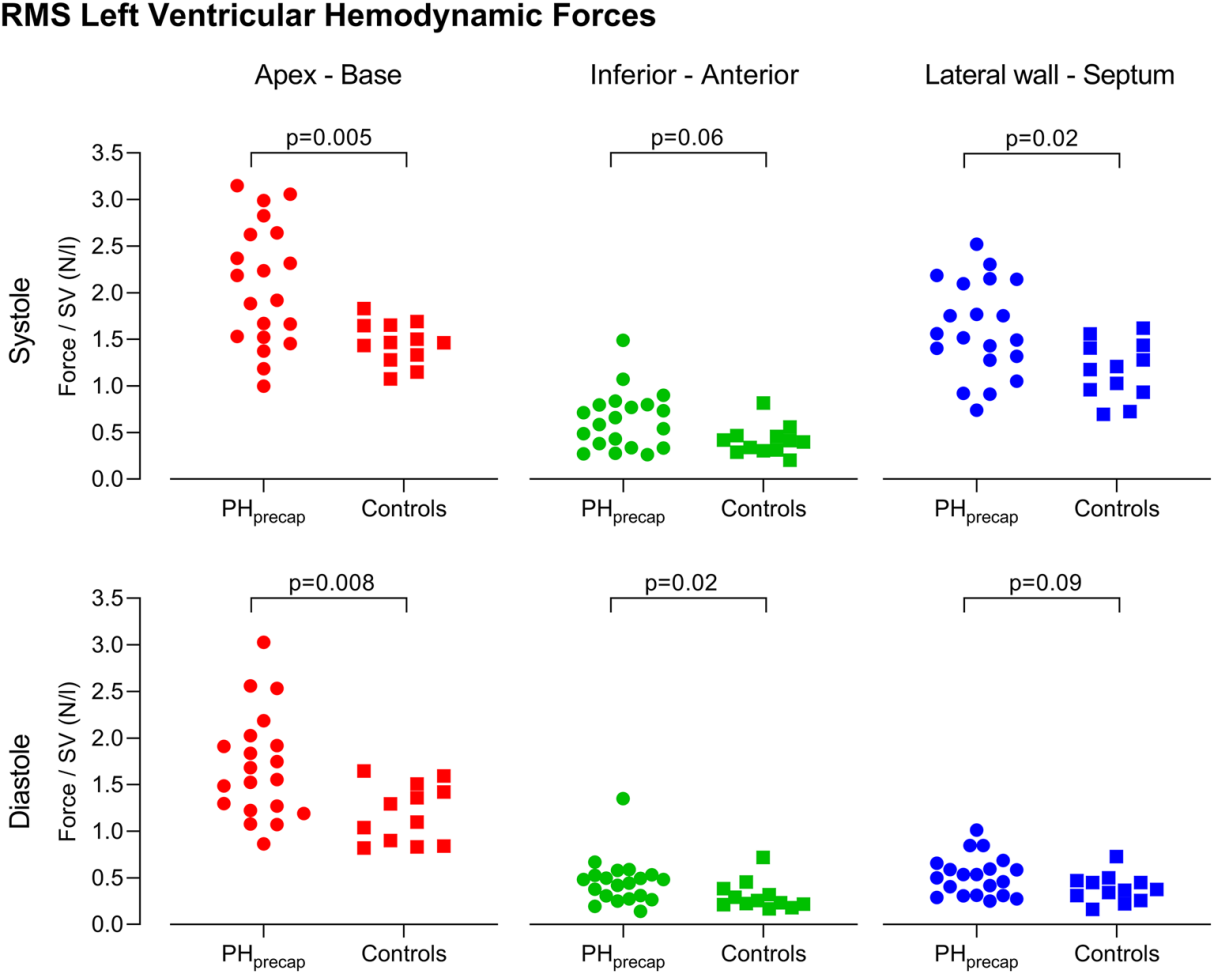
Root mean square (RMS) left ventricular hemodynamic forces in patients with precapillary pulmonary hypertension (PH_precap_, circles) compared to healthy controls (squares). RMS hemodynamic forces indexed to left ventricular stroke volume (SV) during systole (top row) and diastole (bottom row), in the apex-base direction (left column, red), inferior-anterior direction (middle column, green), and lateral wall-septum direction (right column, blue).

LV HDF indexed to SV is presented in Fig. 5 and Supplementary Table 2 for RMS forces and Supplementary Fig. 7 and Supplementary Table 3 for peak forces. Patients had larger HDF (both RMS and peak) than controls in systole in the apex-base and in the lateral wall-septum directions and in diastole in the apex-base and the inferior-anterior directions.

#### LV hemodynamic forces in absolute values

LV HDF in absolute values is presented in Supplementary Fig. 8 and Supplementary Table 4 for RMS forces and Supplementary Fig. 9 and Supplementary Table 5 for peak forces. No differences were found between the groups in any direction for RMS or peak values (p > 0.3 for all).

#### LV force ratio

The ratio between LV transverse and longitudinal hemodynamic forces did not differ between groups in systole or diastole (p = 0.9 for both).

### Hemodynamic forces and stroke volume

Flow volumes in 4D flow was validated in patients with PH_precap_ and healthy controls with 2D flow as the reference standard to ensure sufficient data quality (Appendix). Associations between HDF and SV are presented in Table 3. In patients, biventricular HDF were associated with SV in all three directions in both systole and diastole. In controls, HDF were associated with SV in all three directions in systole in both the RV and LV, but not in diastole.

**Table 3:** Hemodynamic forces and stroke volume.

	PH_precap_ (n = 20) R^2^ (p-value)	Controls (n = 12) R^2^ (p-value)
**Right ventricle**		
Apex-Base, systole	0.3 (*0.01*)	0.7 (*0.001*)
Apex-Base, diastole	0.6 (*0.0001*)	0.3 (0.09)
Diaphragm-Outflow tract, systole	0.4 (*0.005*)	0.5 (*0.01*)
Diaphragm-Outflow tract, diastole	0.5 (*0.0004*)	0.07 (0.4)
Septum-Free wall, systole	0.4 (*0.005*)	0.4 (*0.04*)
Septum-Free wall, diastole	0.5 (*0.0002*)	0.2 (0.2)
**Left ventricle**		
Apex-Base, systole	0.5 (*0.0009*)	0.8 (*0.0002*)
Apex-Base, diastole	0.6 (*0.0001*)	0.2 (0.2)
Inferior-Anterior, systole	0.4 (*0.002*)	0.4 (*0.02*)
Inferior-Anterior, diastole	0.4 (*0.002*)	0.08 (0.4)
Lateral wall-Septum, systole	0.8 (*0.0001*)	0.4 (*0.02*)
Lateral wall-Septum, diastole	0.4 (*0.002*)	0.05 (0.5)

Significant values are in [italics].

Association between systolic root mean square hemodynamic forces (HDF) and stroke volume in patients with PH_precap_ and healthy controls. For R^2^, the independent variable was set as stroke volume, and the dependent variable was set as hemodynamic forces.

Comparing patients with RV EF < 50% (n = 15) to patients with RV EF ≥ 50% (n = 5), patients with RV EF < 50% had increased RV transverse HDF/SV (Table 4). Patients with LV EF < 50% (n = 7) had increased LV HDF/SV compared to patients with LV EF ≥ 50% (n = 13) in the apex-base direction in systole and in the lateral wall-septum direction in diastole (Table 5).

**Table 4:** Right ventricular hemodynamic forces indexed to stroke volume (N/l) for patients with PH_precap_ grouped by ejection fraction.

	PH_precap_ RV EF < 50% (n = 15)	PH_precap_ RV EF ≥ 50% (n = 5)	p-value
**Right ventricle**			
Apex-Base, systole	1.2 [0.54]	1.0 [0.15]	0.3
Apex-Base, diastole	1.5 [0.75]	1.3 [0.28]	0.2
Diaphragm-Outflow tract, systole	2.1 [0.59]	1.1 [0.57]	*0.01*
Diaphragm-Outflow tract, diastole	0.90 [0.37]	0.55 [0.14]	*0.04*
Septum-Free wall, systole	0.86 [0.64]	0.53 [0.18]	*0.0009*
Septum-Free wall, diastole	0.60 [0.50]	0.39 [0.22]	0.1

Significant values are in [italics].

Root mean square right ventricular (RV) hemodynamic forces indexed to RV stroke volume (SV) in patients with precapillary pulmonary hypertension (PH_preap_) and controls. Data is expressed as median [IQR].

**Table 5:** Left ventricular hemodynamic forces indexed to stroke volume (N/l) for patients with PH_precap_ grouped by ejection fraction.

	PH_precap_ LV EF < 50% (n = 7)	PH_precap_ LV EF ≥ 50% (n = 13)	p-value
**Left ventricle**			
Apex-Base, systole	2.4 [0.70]	1.7 [0.86]	*0.03*
Apex-Base, diastole	1.7 [0.83]	1.5 [0.64]	0.5
Inferior-Anterior, systole	0.80 [0.44]	0.54 [0.40]	0.1
Inferior-Anterior, diastole	0.48 [0.12]	0.42 [0.27]	0.7
Lateral wall-Septum, systole	1.8 [0.57]	1.5 [0.82]	0.7
Lateral wall-Septum, diastole	0.69 [0.25]	0.42 [0.23]	*0.005*

Significant values are in [italics].

Root mean square left ventricular (LV) hemodynamic forces indexed to LV stroke volume (SV) in patients with precapillary pulmonary hypertension (PH_preap_) and controls. Data is expressed as median [IQR].

There was no difference in RV or LV HDF/SV in any direction in systole or diastole between patients with cardiac output above or below median (p > 0.09 for all).

### Hemodynamic forces and pulmonary vascular resistance

In patients with PH_precap_, there was no correlation between pulmonary vascular resistance (PVR) and RV systolic RMS or peak HDF in the diaphragm-outflow tract direction in absolute values (RMS: r = − 0.3, p = 0.2; peak: r = − 0.3, p = 0.2) or indexed to RV SV (RMS: r = 0.02, p = 0.9; peak: r = 0.02, p = 0.9).

## Discussion

This study investigated biventricular hemodynamic forces in patients with precapillary pulmonary hypertension and healthy controls. Patients showed larger biventricular hemodynamic forces indexed to SV in all three spatial directions, indicating biventricular pathology both in pumping and filling mechanisms which cannot be explained merely by differences in blood volume. Biventricular hemodynamic force analysis may be a more sensitive marker of pathological cardiac pumping mechanisms than typical volumetric and functional parameters in patients with PH_precap_, and has the potential to improve guidance of treatment effect in this patient cohort.

### Cardiac pumping physiology

#### Pulmonary arterial hypertension and chronic thromboembolic pulmonary hypertension

Pulmonary arterial hypertension is considered to be caused primarily by vascular remodeling of the pulmonary arteries, whereas chronic thromboembolic pulmonary hypertension is mainly associated with pulmonary artery obstructions. Although the etiology differs between the two groups, the pathophysiological effects on the cardiac pumping mechanisms are similar^1^. Septal flattening typical for patients with PH_precap_ was seen in our patient cohort, which may affect hemodynamic forces in both the RV and LV.

#### RV systolic HDF

Patients had larger RV systolic HDF indexed to SV compared to controls in the two transverse directions, indicating that a larger amount of force per ml of blood volume is required in these directions to achieve RV SV. Decreased RV atrioventricular plane displacement (AVPD) and RV longitudinal strain, together with increased transverse forces in patients may indicate that to overcome the increased pulmonary arterial pressure and preserve RV SV, an increased RV contractile drive is mainly obtained in the transverse directions. In the healthy ventricle, the myocardial fibers are arranged in counter-wound helices, allowing a twisting motion during systole^36–42^. Ventricular hypertrophy or dilatation may alter the myocardial structure^43–45^, leading to changes in ventricular motion and thereby affecting the intraventricular blood flow. We found no difference between patients and controls in systolic HDF indexed to SV in the apex-base direction. In the hearts of healthy subjects, SV is primarily driven by AVPD^34^. Patients with PH_precap_ and RV dilatation due to volume overload typically have preserved longitudinal contribution to SV through increased short-axis area, despite decreased tricuspid valve motion^13^. In the compensated stage, patients with PH_precap_ have preserved RV SV and cardiac output^46^, and our data with preserved RV SV in patients could explain the similarity in systolic force patterns in the RV apex-base direction between patients and controls, despite patients having decreased RV EF and RV AVPD. There were no correlations between HDF and pulmonary vascular resistance, indicating that HDF is a measure of intraventricular hemodynamics independent of variations in afterload.

#### RV diastolic HDF

Our data shows that patients had larger RV diastolic hemodynamic forces indexed to SV than controls in all three spatial directions. Filling of the RV in healthy hearts is mainly achieved by moving the AV plane towards the atrium, thus lengthening the ventricle and shifting the position of the tricuspid valve in relation to the blood^34,47,48^. It has been shown that patients with PH_precap_ have a decreased RV isovolumetric relaxation time, filling rate and AVPD, with a larger contribution of atrial contraction compared to controls^13,49^. Pathological dilatation with an abnormally stretched myocardium of the RV in our patient cohort is shown by increased end-diastolic (EDV) and end-systolic (ESV) volumes. Increased diastolic forces and RV EDV but with a preserved RV SV could indicate an increased ventricular stiffness and decreased ventricular compliance, where a larger amount of intraventricular blood is accelerated compared to controls.

#### LV systolic HDF

Systolic HDF indexed to SV were increased in patients with PH_precap_ compared to controls in the LV apex-base and lateral wall-septum directions. These two directions constitute the main direction of systolic blood flow in the healthy heart, as the lateral-wall septum direction is aligned parallel to the LV outflow tract^7,16,50^. Increased systolic longitudinal HDF and reduced AVPD in our patient cohort indicate an altered pumping mechanism to achieve SV. Longitudinal LV function has previously been found reduced in patients with pulmonary hypertension despite preserved LV EF^4,13,14,51^. Our study indicates that patients with PH_precap_ have greater variation in LV pumping mechanisms compared to controls, as systolic force indexed to SV in the LV had a larger range in patients than controls in all three directions. This is in line with previously shown heterogeneity in LV myocardial contractility in patients with PH_precap_, when using myocardial strain as a measurement of deformation^14^. On the group level, patients with PH_precap_ had decreased LV SV and EDV compared to controls, indicating underfilling of the LV. Increased heart rate in patients suggest sympathetic upregulation due to underfilling, which may lead to increased LV contractility and thereby increased LV HDF/SV.

Increased HDF in the LV apex-base and lateral wall-septum directions could be linked to LV anatomical deformation, as increased pressure in the RV leads to the septum bulging into the LV during systole^52,53^. Our data indicate that the anatomical deformations are mainly compensated for in the LV by alterations in fluid dynamics in the apex-base direction. However, the ratio of systolic transverse to longitudinal LV forces was preserved in patients compared to controls. Patients had increased HDF both in the lateral wall-septum and apex-base directions, and together with a preserved force ratio, this indicates that HDF in these two directions are proportionally increased in patients compared to controls.

#### LV diastolic HDF

According to our data, smaller LV EDV does not seem to lead to distinct alterations in absolute values of LV HDF, but LV HDF were larger in patients than controls when indexed to SV. Increased resistance in the pulmonary circulation leads to a decreased filling of the LV in PH_precap_^2^, and as a consequence the LV volumes are smaller than in healthy controls. In our study, underfilling is evident from decreased LV AVPD and LV EDV in patients. The above indicate that HDF is not a substitutional measurement merely for cardiac volumes, but a measurement reflecting physiological mechanisms of the myocardium and the impact on intraventricular blood flow patterns. A recent study has shown reduced LV diastolic intraventricular pressure gradients in PH_precap_ using echocardiography, and this parameter was suggested as a marker of impaired LV suction in this patient group^54^. In contrast, another study suggested that the mechanism causing reduced LV longitudinal function in PAH is underfilling of the LV rather than an intrinsic ventricular dysfunction^4^. The combination of elevated diastolic HDF in the apex-base direction, no difference in LV longitudinal strain between PH_precap_ and controls and decreased LV volumes in our data fits best with LV underfilling in PH_precap_.

### Indexing to SV

Indexing HDF to SV facilitates functional comparison between hearts of different sizes. As force is proportional to mass and acceleration, indexing HDF to SV diminishes the relevance of volumetric differences, and the acceleration component attains greater importance.

In the healthy heart, systolic forces should increase proportionally to SV, as force equals mass multiplied by acceleration (Newton’s second law of motion). Increased HDF/SV indicates a larger acceleration of blood, which in turn likely follows from sympathetic upregulation with increased blood pressure to maintain cardiac output. The same principle applies in diastole, where the filling of the ventricle should equal stroke volume in the healthy heart, according to the Frank-Starling law.

Ventricular volumes play an important role in determining HDF, as a previous study has shown that HDF analysis cannot separate heart-failure patients with preserved EF from controls, but patients with impaired ventricular volumes were found to have impaired volume-normalized HDF^18^. We similarly saw differences between patients with PH_precap_ and controls when indexing HDF to SV.

Stroke volume differed between patients and controls in the LV, but not in the RV. When quantifying RMS HDF in absolute values, differences between the groups were found in one direction in the RV and none of the directions in the LV (Supplementary Figs. 4, 5, 8 and 9). However, when indexing HDF to SV, differences were found between the groups in all three directions both in the RV and LV. In patients, biventricular HDF were associated with SV in systole and diastole in all three directions. In controls, associations between biventricular HDF and SV were found in systole in all three directions, but not in diastole. As SV should equal diastolic filling volume, this indicates that patients with PH_precap_ have an altered relation between force and diastolic filling compared to healthy controls.

### Comparison to earlier studies

Previous studies have shown that healthy volunteers, athletes and patients with heart failure with preserved ejection fraction tend to have homogeneous HDF patterns, whereas HDF in heart diseases with abnormal ventricular volumes, impaired systolic function, or significant valvular insufficiencies such as dilated cardiomyopathy, heart failure with left ventricular dyssynchrony, and repaired tetralogy of Fallot, generally show more heterogeneous patterns^15,16,18,55^. Alterations in HDF have primarily been found in diastole in patients with left-sided heart failure, which was also true in our study^55,56^.

A recent study by Vos et al.^57^ has analyzed LV pressure gradients normalized to LV volumes in the apex-base direction in patients with PH_precap_. Their patient cohort had reduced LV EDV and preserved LV EF, which is in line with our data. Interestingly, in the study by Vos et al. patients had preserved LV pressure gradients in systole and decreased in diastole, which contradicts our results where patients had increased LV HDF in systole and diastole. In the study by Vos et al., the authors interpret the results as impaired diastolic function of the LV. However, another explanation for altered pressure gradients could be underfilling of the LV since the patient cohorts in both studies have reduced LV volumes, although this does not explain the discrepancy of the results in these two studies.

There are methodological differences in the study by Vos et al. compared to our study, which could be a part of the explanation of the contradictory results^12^. Vos et al. use a numerical approach based on feature-tracking of cine images for calculation of pressure gradients, whereas 4D flow is used in our study. Furthermore, there are differences in the patient cohorts in our study and the study by Vos et al*.*, where 30% of our patients had PH-specific medication at CMR, compared to 97% in the study by Vos et al. Another difference is that our patients and controls were matched for age and sex, whereas the groups in the aforementioned study were not, and instead their data were adjusted statistically for age and sex.

Previously, differences in HDF have been shown in patients with dilated cardiomyopathy compared to controls^16^. In patients with PH_precap_, typically the RV is dilated, and LV volumes are reduced. Our data show that HDF are more affected in the RV than in the LV in this patient cohort, which is in line with previous data of alterations in HDF in the dilated ventricle.

Patients with heart failure and preserved LV EF have been shown to have lower LV hemodynamic longitudinal forces per blood volume compared to controls^8^, although measured with a cine feature-tracking model, which has been shown less sensitive than our method based on 4D flow^12^. Our group of patients has preserved LV EF, but larger forces per blood volume than controls in this direction. Patients with PH_precap_ tend to have smaller LV SV, EDV and ESV. Even if the LV function is impaired, upregulation of the sympathetic system to maintain the pressure can enable preservation of EF, and may result in increased acceleration of the blood. Hemodynamic force analysis has the potential to be a more sensitive marker of cardiac deterioration than typical volumetric and functional parameters when evaluating treatment effect, as patients had increased HDF/SV despite similar LV EF and CO compared to controls.

### Limitations

4D flow measurements could have lower accuracy and precision in patients with right heart failure than controls due to strenuous breathing and increased gross body motion. However, our validation of 4D-flow data to 2D-flow data showed good agreement of net flow between 4D and 2D flow in both patients with PH_precap_ and healthy controls. 4D flow can therefore be used for assessment of blood flow in patients with PH_precap_.

This study included a small number of patients. However, distinct differences in HDF were found between patients and controls. A subgroup of patients (n = 3) had right or left bundle branch block, which may affect the interpretation of the results for hemodynamic forces. However, ventricular dyssynchrony mainly affects the force ratio^55,56^, which did not differ between patients and controls in the RV or LV in our study. The effects of different loading conditions on HDF are currently unexplored, and the biological variability of HDF within the same individual over time is likewise poorly characterized. In future studies, a larger number of patients is of interest, for analysis of diagnostic subgroups and of patients in different stages of heart failure. The prognostic and therapeutical implications also merit further study, as well as HDF in serial examinations for investigation of HDF from the compensated to the uncompensated stage of heart failure.

## Conclusion

Hemodynamic force analysis conveys information of pathological cardiac pumping mechanisms complementary to more established volumetric and functional parameters in precapillary pulmonary hypertension. The right ventricle compensates for the increased afterload in part by augmenting transverse forces, and left ventricular hemodynamic abnormalities are mainly a result of underfilling rather than intrinsic ventricular dysfunction.

## Supplementary Information

Supplementary Information.

## Data Availability

The data in this study is available from the corresponding author on reasonable request. The analysis software Segment used to analyze CMR data is freely available.

## References

[1] Galiè N (2016). 2015 ESC/ERS Guidelines for the diagnosis and treatment of pulmonary hypertension. Eur. Heart J..

[2] Van Wolferen SA (2007). Prognostic value of right ventricular mass, volume, and function in idiopathic pulmonary arterial hypertension. Eur. Heart J..

[3] Addetia K (2016). Three-dimensional echocardiography-based analysis of right ventricular shape in pulmonary arterial hypertension. Eur. Heart J. Cardiovasc. Imaging.

[4] Sjögren H (2021). Underfilling decreases left ventricular function in pulmonary arterial hypertension. Int. J. Cardiovasc. Imaging.

[5] Bredfelt A, Rådegran G, Hesselstrand R, Arheden H, Ostenfeld E (2018). Increased right atrial volume measured with cardiac magnetic resonance is associated with worse clinical outcome in patients with pre-capillary pulmonary hypertension. ESC Hear. Fail..

[6] Dong Y (2020). Prognostic value of cardiac magnetic resonance—derived right ventricular remodeling parameters in pulmonary hypertension. Circ. Cardiovasc. Imaging.

[7] Arvidsson PM (2017). Left and right ventricular hemodynamic forces in healthy volunteers and elite athletes assessed with 4D flow magnetic resonance imaging. Am. J. Physiol. Hear. Circ. Physiol..

[8] Lapinskas T (2019). The intraventricular hemodynamic forces estimated using routine CMR cine images: A new marker of the failing heart. JACC Cardiovasc. Imaging.

[9] Vallelonga F (2021). Introduction to hemodynamic forces analysis: Moving into the new frontier of cardiac deformation analysis. J. Am. Heart Assoc..

[10] Pedrizzetti G (2016). Changes in electrical activation modify the orientation of left ventricular flow momentum: Novel observations using echocardiographic particle image velocimetry. Eur. Heart J. Cardiovasc. Imaging.

[11] Töger J (2018). Hemodynamic forces in the left and right ventricles of the human heart using 4D flow magnetic resonance imaging: Phantom validation, reproducibility, sensitivity to respiratory gating and free analysis software. PLoS ONE.

[12] Pedrizzetti G (2017). On estimating intraventricular hemodynamic forces from endocardial dynamics: A comparative study with 4D flow MRI. J. Biomech..

[13] Ostenfeld E (2016). Regional contribution to ventricular stroke volume is affected on the left side, but not on the right in patients with pulmonary hypertension. Int. J. Cardiovasc. Imaging.

[14] Hardegree EL (2013). Impaired left ventricular mechanics in pulmonary arterial hypertension—identification of a cohort at high risk. Circ. Hear. Fail..

[15] Sjöberg P (2018). Altered biventricular hemodynamic forces in patients with repaired tetralogy of fallot and right ventricular volume overload because of pulmonary regurgitation. Am. J. Physiol. Hear. Circ. Physiol..

[16] Eriksson J, Bolger AF, Ebbers T, Carlhäll CJ (2016). Assessment of left ventricular hemodynamic forces in healthy subjects and patients with dilated cardiomyopathy using 4D flow MRI. Physiol. Rep..

[17] Pedrizzetti G, Faganello G, Croatto E, Di Lenarda A (2021). The hemodynamic power of the heart differentiates normal from diseased right ventricles. J. Biomech..

[18] Arvidsson PM (2022). Hemodynamic force analysis is not ready for clinical trials on HFpEF. Sci. Rep..

[19] Bergström G (2015). The Swedish CArdioPulmonary bioimage study: Objectives and design. J. Intern. Med..

[20] Rothwell A (2007). Strengthening the reporting of observational studies in epidemiology (STROBE) statement : Guidelines for reporting observational studies. Br. Med. J..

[21] Stefadouros MA, Dougherty MJ, Grossman W, Craige E (1973). Determination of systemic vascular resistance by a noninvasive technic. Circulation.

[22] Feinstein JA (1997). Using cardiac phase to order reconstruction (CAPTOR): A method to improve diastolic images. J. Magn. Reson. Imaging.

[23] Dyverfeldt P (2015). 4D flow cardiovascular magnetic resonance consensus statement. J. Cardiovasc. Magn. Reson..

[24] Kanski M (2015). Whole-heart four-dimensional flow can be acquired with preserved quality without respiratory gating, facilitating clinical use: A head-to-head comparison. BMC Med. Imaging.

[25] Carlsson M (2011). Quantification and visualization of cardiovascular 4D velocity mapping accelerated with parallel imaging or k-t BLAST: Head to head comparison and validation at 1.5 T and 3 T. J. Cardiovasc. Magn. Reson..

[26] Töger J (2016). Independent validation of four-dimensional flow MR velocities and vortex ring volume using particle imaging velocimetry and planar laser-Induced fluorescence. Magn. Reson. Med..

[27] Busch J, Giese D, Kozerke S (2017). Image-based background phase error correction in 4D flow MRI revisited. J. Magn. Reson. Imaging.

[28] Gatehouse P (2010). Flow measurement by cardiovascular magnetic resonance: A multi-centre multi-vendor study of background phase offset errors that can compromise the accuracy of derived regurgitant or shunt flow measurements. J. Cardiovasc. Magn. Reson..

[29] Yang GZ, Burger P, Kilner PJ, Karwatowski SP, Firmin DN (1996). Dynamic range extension of cine velocity measurements using motion-registered spatiotemporal phase unwrapping. J. Magn. Reson. Imaging.

[30] Bock J (2019). Validation and reproducibility of cardiovascular 4D-flow MRI from two vendors using 2 × 2 parallel imaging acceleration in pulsatile flow phantom and in vivo with and without respiratory gating. Acta radiol..

[31] Heiberg E (2010). Design and validation of Segment—freely available software for cardiovascular image analysis. BMC Med. Imaging.

[32] Schulz-Menger J (2013). Standardized image interpretation and post processing in cardiovascular magnetic resonance: Society for Cardiovascular Magnetic Resonance (SCMR) Board of Trustees Task Force on Standardized Post Processing. J. Cardiovasc. Magn. Reson..

[33] Carlsson M, Ugander M, Heiberg E, Arheden H (2007). The quantitative relationship between longitudinal and radial function in left, right, and total heart pumping in humans. Am. J. Physiol. Hear. Circ. Physiol..

[34] Carlsson M, Ugander M, Mosén H, Buhre T, Arheden H (2007). Atrioventricular plane displacement is the major contributor to left ventricular pumping in healthy adults, athletes, and patients with dilated cardiomyopathy. Am. J. Physiol. Hear. Circ. Physiol..

[35] Poms AD, Turner M, Farber HW, Meltzer LA, McGoon MD (2013). Comorbid conditions and outcomes in patients with pulmonary arterial hypertension: A reveal registry analysis. Chest.

[36] Shapiro EP, Rademakers FE (1997). Importance of oblique fiber orientation for left ventricular wall deformation. Technol. Heal. Care.

[37] Ingels NB (1997). Myocardial fiber architecture and left ventricular function. Technol. Heal. Care.

[38] Buckberg GD, Coghlan HC, Torrent-Guasp F (2001). The structure and function of the helical heart and its buttress wrapping. V. Anatomic and physiologic considerations in the healthy and failing heart. Semin. Thorac. Cardiovasc. Surg..

[39] Buckberg GD (2002). Basic science review: The helix and the heart. J. Thorac. Cardiovasc. Surg..

[40] Coghlan C, Hoffman J (2006). Leonardo da Vinci’s flights of the mind must continue: Cardiac architecture and the fundamental relation of form and function revisited. Eur. J. Cardio-thoracic Surg..

[41] Sallin EA (1969). Fiber orientation and ejection fraction in the human left ventricle. Biophys. J..

[42] Pettigrew JB (1864). On the arrangement of the muscular fibres in the ventricles of the vertebrate heart, with physiological remarks. Philos. Trans. R. Soc. Lond..

[43] Weber KT (1990). Fibrillar collagen and remodeling of dilated canine left ventricle. Circulation.

[44] Stuber M (1999). Alterations in the local myocardial motion pattern in patients suffering from pressure overload due to aortic stenosis. Circulation.

[45] Weber KT, Jalil JE, Janicki JS, Pick R (1989). Myocardial collagen remodeling in pressure overload hypertrophy. Am. J. Hypertens..

[46] Marcus JT (2001). Impaired left ventricular filling due to right ventricular pressure overload in primary pulmonary hypertension. Chest.

[47] Brecher GA (1956). Experimental evidence of ventricular diastolic suction. Circ. Res..

[48] Yellin EL, Nikolic S, Frater RWM (1990). Left ventricular filling dynamics and diastolic function. Prog. Cardiovasc. Dis..

[49] Gan CTJ (2007). Right ventricular diastolic dysfunction and the acute effects of sildenafil in pulmonary hypertension patients. Chest.

[50] Pedrizzetti G (2015). Cardiac fluid dynamics anticipates heart adaptation. J. Biomech..

[51] Lindholm A, Hesselstrand R, Rådegran G, Arheden H, Ostenfeld E (2019). Decreased biventricular longitudinal strain in patients with systemic sclerosis is mainly caused by pulmonary hypertension and not by systemic sclerosis per se. Clin. Physiol. Funct. Imaging.

[52] Boxt LM, Katz J, Kolb T, Czegledy FP, Barst RJ (1992). Direct quantitation of right and left ventricular volumes with nuclear magnetic resonance imaging in patients with primary pulmonary hypertension. J. Am. Coll. Cardiol..

[53] Bristow MR (1998). The pressure-overloaded right ventricle in pulmonary hypertension. Chest.

[54] Chiba Y (2022). Determinants of altered left ventricular suction in pre-capillary pulmonary hypertension. Eur. Hear. J. Cardiovasc. Imaging.

[55] Arvidsson PM (2018). Hemodynamic forces using four-dimensional flow MRI: An independent biomarker of cardiac function in heart failure with left ventricular dyssynchrony?. Am. J. Physiol. Hear. Circ. Physiol..

[56] Eriksson J (2017). Left ventricular hemodynamic forces as a marker of mechanical dyssynchrony in heart failure patients with left bundle branch block. Sci. Rep..

[57] Vos, J. L. *et al.* Cardiovascular magnetic resonance-derived left ventricular intraventricular pressure gradients among patients with precapillary pulmonary hypertension. *Eur. Hear. J. Cardiovasc. Imaging.* 1–10 (2022). 10.1093/ehjci/jeab294 34993533

