# Supplementary material for: Increased biventricular hemodynamic forces in precapillary pulmonary hypertension
Source: Sci Rep. 2022 Nov 19;12:19933. doi: 10.1038/s41598-022-24267-6 (PMC9675772; doi:10.1038/s41598-022-24267-6)
Supplement: Supplementary file 1 — Supplementary Information. [file 41598_2022_24267_MOESM1_ESM.docx]

**Supplementary data**

**Increased biventricular hemodynamic forces in precapillary pulmonary hypertension**

K. Pola^1^, E. Bergström^1^, J. Töger^1^, G. Rådegran^2^, P.M. Arvidsson^1^, M. Carlsson^1^, H. Arheden^1^, E. Ostenfeld^1*^

1. Clinical Physiology, Department of Clinical Sciences Lund, Lund University, Skåne University Hospital, Lund, Sweden.

2. Lund University, Department of Clinical Sciences Lund, Cardiology, and Skåne University Hospital, Section of Heart Failure and Valvular Disease, Lund, Sweden

*Corresponding author:
Ellen Ostenfeld
+46 46 173335
[ellen.ostenfeld@med.lu.se](mailto:ellen.ostenfeld@med.lu.se)
Skåne University Hospital
Dept of Clinical Physiology
SE-221 85 Lund, Sweden

**Keywords:** Precapillary pulmonary hypertension, hemodynamic forces, 4D flow, cardiac magnetic resonance, left and right ventricle

**Supplementary Table 1. Typical 4D flow sequence parameters**

| **Parameter** |  |
| --- | --- |
| Readout strategy | Cartesian |
| Gating | Respiratory gating in 20 cases (15 patients, 5 controls), 12 without (5 patients, 7 controls). |
| Velocity encoding (VENC), cm/s | 100 (n=22), 150 (n=6), 250 (n=4) |
| Echo time, ms | 2.8-3.5 |
| Repetition time, ms | 5.8 |
| Flip angle, ° | 15 (n=27, post contrast), 8 (n=5, no contrast) |
| Bandwidth/pixel, Hz | 558 |
| Field of view (mm) | 288 × 240 × 162 |
| Matrix size (readout × phase × slice) | 96 × 80 × 54 |
| Acquired spatial resolution (mm) | 3 × 3 × 3 |
| Reconstructed spatial resolution (mm) | 3 × 3 × 3 |
| Temporal segmentation factor | 2 |
| Acquired temporal resolution (ms) | 46 |
| Reconstructed temporal resolution, ms | 16-29 (40 phases per cardiac cycle) |
| Acceleration methods | GRAPPA (phase × slice 2×2 or 3x1) partial Fourier (phase x slice 6/8 × 6/8) |
| Postprocessing | Maxwell correction, background correction, phase unwrapping. |

**Supplementary Table 2. Biventricular RMS hemodynamic forces.**

|  | **PH_precap_  (n=20)** | **Controls  (n=12)** | **p-value** |
| --- | --- | --- | --- |
| ***Right ventricle, HDF (N/l)*** |  |  |  |
| - Apex-Base, systole | 1.1 [0.93-1.4] | 1.1 [0.87-1.2] | 0.4 |
| - Apex-Base, diastole | 1.4 [1.3-1.9] | 0.87 [0.62-0.99] | *<0.0001* |
| - Diaphragm-Outflow tract, systole | 2.1 [1.6-2.3] | 1.4 [1.1-1.5] | *0.003* |
| - Diaphragm-Outflow tract, diastole | 0.80 [0.54-0.93] | 0.47 [0.29-0.53] | *0.005* |
| - Septum-Free wall, systole | 0.64 [0.56-1.2] | 0.42 [0.24-0.57] | *0.007* |
| - Septum-Free wall, diastole | 0.60 [0.51-0.84] | 0.38 [0.34-0.45] | *0.003* |
| ***Left ventricle, HDF (N/l)*** |  |  |  |
| - Apex-Base, systole | 2.1 [1.5-2.6] | 1.5 [1.3-1.6] | *0.005* |
| - Apex-Base, diastole | 1.6 [1.3-1.9] | 1.2 [0.88-1.4] | *0.008* |
| - Inferior-Anterior, systole | 0.62 [0.37-0.80] | 0.41 [0.31-0.46] | 0.06 |
| - Inferior-Anterior, diastole | 0.46 [0.30-0.53] | 0.24 [0.22-0.34] | *0.02* |
| - Lateral wall-Septum, systole | 1.5 [1.3-2.1] | 1.2 [0.95-1.4] | *0.02* |
| - Lateral wall-Septum, diastole | 0.52 [0.31-0.61] | 0.37 [0.30-0.45] | 0.09 |

Data is expressed as median [IQR]. Root mean square (RMS) hemodynamic forces (HDF) indexed to stroke volume of each ventricle respectively in patients with precapillary pulmonary hypertension (PH_precap_) and healthy controls.

**Supplementary Table 3. Biventricular peak hemodynamic forces.**

|  | **PH_precap_  (n=20)** | **Controls (n=12)** | **p-value** |
| --- | --- | --- | --- |
| ***Right ventricle, HDF (N/l)*** |  |  |  |
| - Apex-Base, systole | 2.4 [1.9-3.2] | 2.0 [1.8-2.3] | 0.1 |
| - Apex-Base, diastole | 3.6 [3.1-4.5] | 1.8 [1.6-2.1] | *<0.0001* |
| - Diaphragm-Outflow tract, systole | 4.4 [3.6-5.3] | 2.8 [2.2-3.2] | *0.0008* |
| - Diaphragm-Outflow tract, diastole | 1.9 [1.1-2.3] | 1.1 [0.64-1.2] | *0.01* |
| - Septum-Free wall, systole | 1.4 [1.2-3.2] | 0.91 [0.50-1.2] | *0.004* |
| - Septum-Free wall, diastole | 1.2 [1.0-1.6] | 0.92 [0.73-1.1] | *0.02* |
| ***Left ventricle, HDF (N/l)*** |  |  |  |
| - Apex-Base, systole | 3.8 [2.8-4.9] | 2.9 [2.5-3.1] | *0.02* |
| - Apex-Base, diastole | 4.2 [2.9-4.6] | 2.5 [2.1-3.1] | *0.002* |
| - Inferior-Anterior, systole | 1.3 [0.98-1.7] | 0.96 [0.71-1.2] | *0.04* |
| - Inferior-Anterior, diastole | 0.93 [0.73-1.4] | 0.64 [0.53-0.69] | *0.02* |
| - Lateral wall-Septum, systole | 2.9 [2.5-3.4] | 2.1 [1.7-2.3] | *0.001* |
| - Lateral wall-Septum, diastole | 1.2 [0.83-1.5] | 0.90 [0.59-1.3] | 0.05 |

Data is expressed as median [IQR]. Peak hemodynamic forces (HDF) indexed to stroke volume of each ventricle respectively in patients with precapillary pulmonary hypertension (PH_precap_) and healthy controls.

**Supplementary Table 4. Biventricular RMS hemodynamic forces in absolute values.**

|  | **PH_precap_**  **(n=20)** | **Controls  (n=12)** | **p-value** |
| --- | --- | --- | --- |
| ***Right ventricle, HDF (N)*** |  |  |  |
| - Apex-Base, systole | 0.078 [0.058-0.097] | 0.089 [0.061-0.11] | 0.7 |
| - Apex-Base, diastole | 0.10 [0.078-0.13] | 0.062 [0.052-0.073] | *0.003* |
| - Diaphragm-Outflow tract, systole | 0.12 [0.10-0.17] | 0.11 [0.090-0.12] | 0.2 |
| - Diaphragm-Outflow tract, diastole | 0.051 [0.032-0.063] | 0.033 [0.026-0.043] | 0.06 |
| - Septum-Free wall, systole | 0.045 [0.033-0.071] | 0.022 [0.017-0.057] | 0.05 |
| - Septum-Free wall, diastole | 0.034 [0.028-0.060] | 0.031 [0.023-0.041] | 0.2 |
| ***Left ventricle, HDF (N)*** |  |  |  |
| - Apex-Base, systole | 0.12 [0.096-0.17] | 0.12 [0.10-0.15] | 1 |
| - Apex-Base, diastole | 0.10 [0.069-0.15] | 0.092 [0.083-0.12] | 0.9 |
| - Inferior-Anterior, systole | 0.042 [0.023-0.057] | 0.032 [0.024-0.042] | 0.7 |
| - Inferior-Anterior, diastole | 0.025 [0.016-0.045] | 0.023 [0.018-0.030] | 0.5 |
| - Lateral wall-Septum, systole | 0.091 [0.070-0.14] | 0.098 [0.075-0.12] | 0.7 |
| - Lateral wall-Septum, diastole | 0.033 [0.021-0.048] | 0.032 [0.024-0.036] | 0.9 |

Data is expressed as median [IQR] root mean square (RMS) hemodynamic forces (HDF) of each ventricle respectively.

**Supplementary Table 5. Biventricular peak hemodynamic forces in absolute values.**

|  | **PH_precap_**  **(n=20)** | **Controls  (n=12)** | **p-value** |
| --- | --- | --- | --- |
| ***Right ventricle, HDF (N)*** |  |  |  |
| - Apex-Base, systole | 0.16 [0.12-0.21] | 0.17 [0.13-0.20] | 1 |
| - Apex-Base, diastole | 0.26 [0.19-0.33] | 0.15 [0.12-0.16] | *0.0004* |
| - Diaphragm-Outflow tract, systole | 0.26 [0.22-0.36] | 0.21 [0.18-0.27] | 0.06 |
| - Diaphragm-Outflow tract, diastole | 0.10 [0.081-0.15] | 0.069 [0.051-0.10] | 0.1 |
| - Septum-Free wall, systole | 0.090 [0.067-0.19] | 0.045 [0.035-0.12] | *0.03* |
| - Septum-Free wall, diastole | 0.079 [0.054-0.12] | 0.077 [0.046-0.096] | 0.4 |
| ***Left ventricle, HDF (N)*** |  |  |  |
| - Apex-Base, systole | 0.22 [0.19-0.30] | 0.25 [0.21-0.28] | 0.9 |
| - Apex-Base, diastole | 0.25 [0.19-0.33] | 0.21 [0.18-0.26] | 0.3 |
| - Inferior-Anterior, systole | 0.087 [0.052-0.15] | 0.071 [0.054-0.11] | 0.7 |
| - Inferior-Anterior, diastole | 0.056 [0.041-0.089] | 0.052 [0.038-0.075] | 0.3 |
| - Lateral wall-Septum, systole | 0.17 [0.13-0.25] | 0.18 [0.13-0.20] | 0.8 |
| - Lateral wall-Septum, diastole | 0.080 [0.053-0.12] | 0.073 [0.054-0.088] | 0.7 |

Data is expressed as median [IQR] peak hemodynamic forces (HDF) of each ventricle respectively.

**
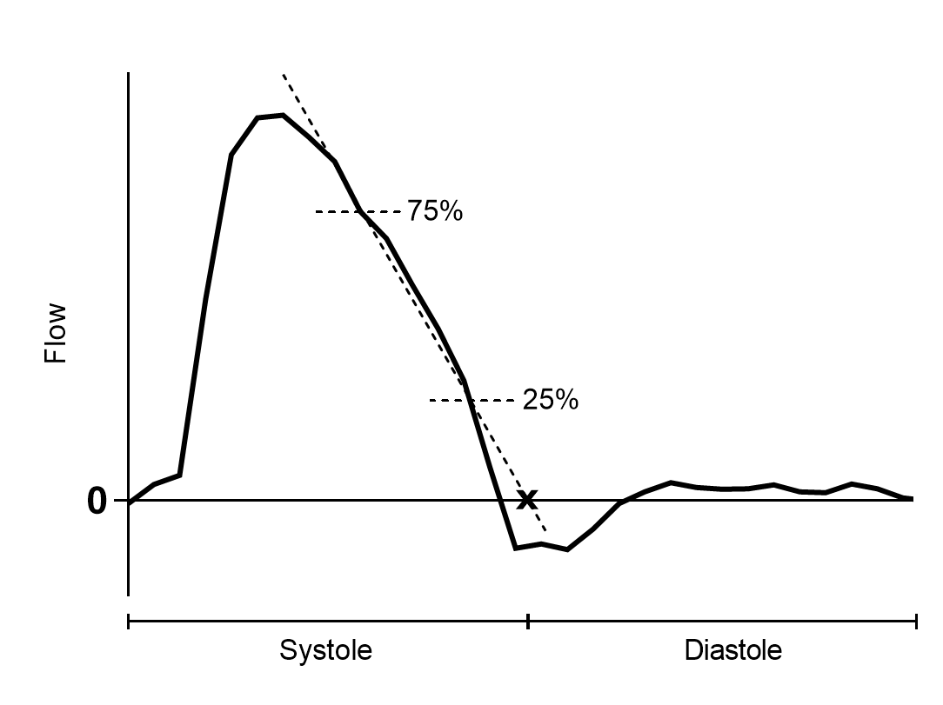
**

**Supplementary Figure 1.** Definition of end systole from flow curves in the aorta (left ventricle) and pulmonary artery (right ventricle). End systole was defined as the timepoint where flow=0 (black cross) on the downslope extrapolated between 75% and 25% of maximum amplitude.

**Supplementary Figure 2. Right ventricular hemodynamic forces over one cardiac cycle.** The force patterns are presented for the patients with precapillary pulmonary hypertension (PH_precap_, left column) and healthy controls (right column), in the apex-base direction (top row, red), diaphragm-outflow tract direction (middle row, green), and septum-free wall direction (bottom row, blue). Mean force curves for each direction are indicated with black lines.

**Supplementary Figure 3. Peak right ventricular hemodynamic forces in patients with precapillary pulmonary hypertension (PH_precap_, circles) compared to healthy controls (squares).** Peak hemodynamic forces indexed to right ventricular stroke volume (SV) during systole (top row) and diastole (bottom row), in the apex-base direction (left column, red), diaphragm-outflow tract direction (middle column, green), and septum-free wall direction (right column, blue).

**Supplementary Figure 4. Root mean square (RMS) right ventricular hemodynamic forces in patients with precapillary pulmonary hypertension (PH_precap_, circles) compared to healthy controls (squares).** RMS hemodynamic forces during systole (top row) and diastole (bottom row), in the apex-base direction (left column, red), diaphragm-outflow tract direction (middle column, green), and septum-free wall direction (right column, blue).

**Supplementary Figure 5. Peak right ventricular hemodynamic forces in patients with precapillary pulmonary hypertension (PH_precap_, circles) compared to healthy controls (squares).** Peak hemodynamic forces during systole (top row) and diastole (bottom row), in the apex-base direction (left column, red), diaphragm-outflow tract direction (middle column, green), and septum-free wall direction (right column, blue).

**Supplementary Figure 6. Left ventricular hemodynamic forces over one cardiac cycle.** The force patterns are presented for the patients with precapillary pulmonary hypertension (PH_precap_, left column) and healthy controls (right column), in the apex-base direction (top row, red), inferior-anterior direction (middle row, green), and lateral wall-septum direction (bottom row, blue). Mean force curves for each direction are indicated with black lines.

**Supplementary Figure 7.** **Peak left ventricular hemodynamic forces in patients with precapillary pulmonary hypertension (PH_precap_, circles) compared to healthy controls (squares).** Peak hemodynamic forces indexed to left ventricular stroke volume (SV) during systole (top row) and diastole (bottom row), in the apex-base direction (left column, red), inferior-anterior direction (middle column, green), and lateral wall-septum direction (right column, blue).

**Supplementary Figure 8.** **Root mean square (RMS) left ventricular hemodynamic forces in patients with precapillary pulmonary hypertension (PH_precap_, circles) compared to healthy controls (squares).** RMS hemodynamic forces during systole (top row) and diastole (bottom row), in the apex-base direction (left column, red), inferior-anterior direction (middle column, green), and lateral wall-septum direction (right column, blue).

**Supplementary Figure 9. Peak left ventricular hemodynamic forces in patients with precapillary pulmonary hypertension (PH_precap_, circles) compared to healthy controls (squares).** Peak hemodynamic forces during systole (top row) and diastole (bottom row), in the apex-base direction (left column, red), inferior-anterior direction (middle column, green), and lateral wall-septum direction (right column, blue).

Appendix

This appendix describes the validation of 4D flow measurements to reference 2D flow in patients with PH_precap_ and healthy subjects.

Methods

Patients with PH_precap_ (n=20) and age- and sex matched healthy controls (n=12) underwent CMR using 1.5T MAGNETOM Aera (Siemens Healthcare, Erlangen, Germany). 4D-flow images were acquired using a prototype sequence. 2D-flow planes were positioned perpendicular to the main pulmonary artery, ascending and descending aorta, right inferior pulmonary vein and superior vena cava (Appendix Figure 1). Regions of interest were delineated in the software Segment v2.2 R7052 (Medviso, Lund, Sweden) (1) in 2D images and delineations were transferred to 4D images. Net flow during one cardiac cycle was computed from both 4D and 2D flow in all delineated vessels. Data from 19 measurements were excluded due to inadequate 4D or 2D flow data quality. Agreement between the methods was assessed using Pearson correlations, modified Bland-Altman analyses, and coefficient of variation in the patient group and control group.

Results

Net flow from 4D flow agreed with 2D flow in patients with PH_precap_ and in healthy controls (r≥0.95, p<0.0001 for both) with low bias (≤(-4.5) ml 4D vs 2D) for patients and for controls (Appendix Table 1 and Appendix Figure 2). Coefficient of variation was 18% for patients and 16% for controls (Appendix Table 1).

Conclusions

There was good agreement of net flow between 4D and 2D flow in both patients with PH_precap_ and healthy controls. 4D flow can therefore be used for assessment of blood flow in patients with PH_precap_.

References

1. Heiberg, E. et al. Design and validation of Segment - freely available software for cardiovascular image analysis. BMC Med. Imaging 10, 1–13 (2010).

**Appendix Table 1.** Difference in net flow between 4D and 2D flow in patients with precapillary pulmonary hypertension (PH_precap_) and healthy controls.

|  | **PH_precap_  (n=20)** | **Controls (n=12)** |
| --- | --- | --- |
| Pearson r | 0.95 | 0.95 |
| Bias±SD | -3.6±7.8 ml | -4.0±8.2 ml |
| Upper limit of agreement (95%) | 12 ml | 12 ml |
| Lower limit of agreement (95%) | -19 ml | -20 ml |
| Mean 2D flow, all vessels | 44 ml | 47 ml |
| Coefficient of variation | 18% | 17% |

**
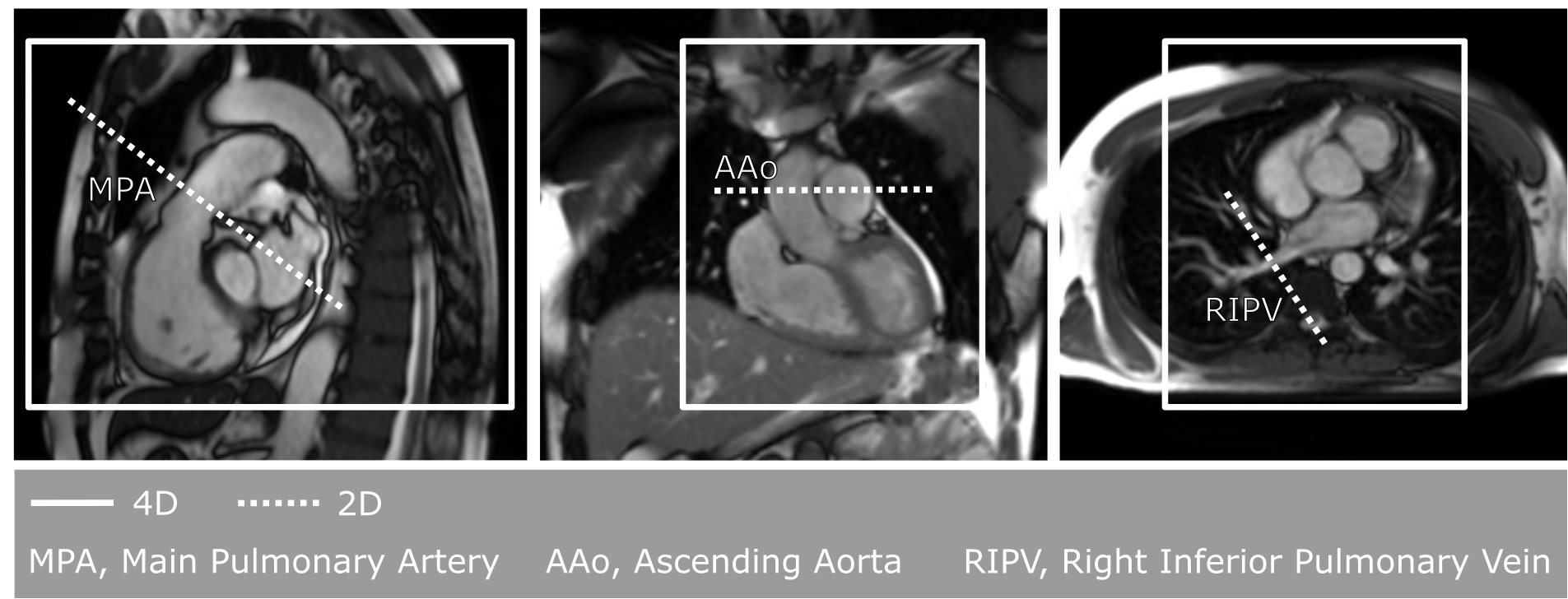
Appendix Figure 1.** Visualization of how the 4D-flow box (full lines) and 2D flow images (dotted lines) relate to each other.


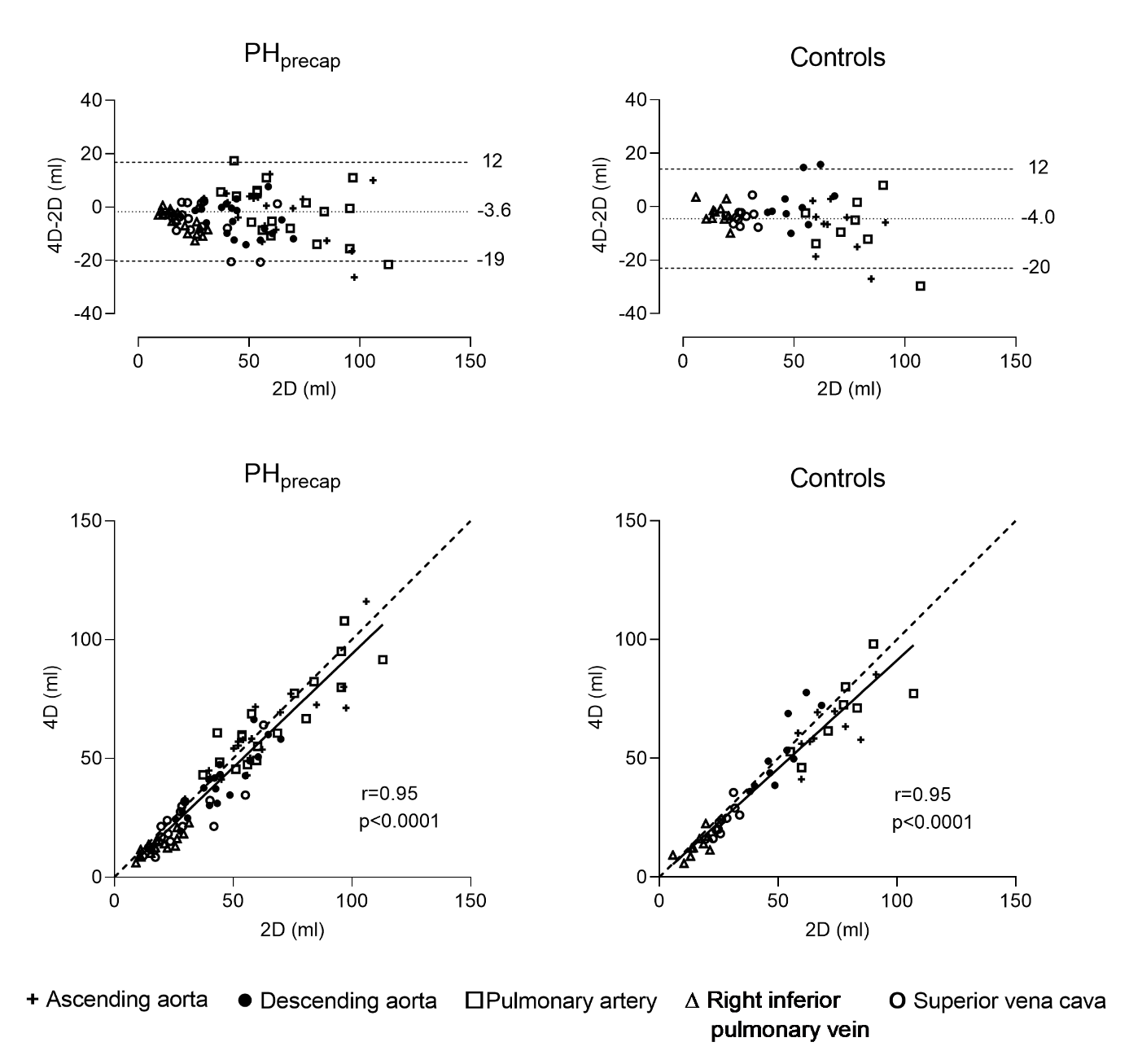
**Appendix Figure 2. Modified Bland-Altman plots and scatter plots of 4D vs 2D flow.** *Upper panel:* Modified Bland-Altman plots with bias±2SD of 4D vs 2D flow, in patients with precapillary pulmonary hypertension (PH_precap_, left) and healthy controls (right). *Lower panel:* Scatter plots of 4D flow vs 2D flow. Dashed line is the line of identity and solid line Pearson’s correlation.
